# Addressing Under-Detection in Minimum Data Set Behavioral Measures Using NIH Stage III/IV Embedded Trial Design

**DOI:** 10.1093/geroni/igaa057.1570

**Published:** 2020-12-16

**Authors:** Ellen McCreedy, Roee Gutman, James Rudolph, Rosa Baier, Kali Thomas, Faye Dvorchak, Jessica Ogarek, Vincent Mor

**Affiliations:** 1 Brown University, Providence, Rhode Island, United States; 2 U.S. Department of Veterans Affairs Medical Center, Providence, Rhode Island, United States; 3 Center for Long-Term Care Quality & Innovation, Providence, Rhode Island, United States; 4 Brown University, PROVIDENCE, Rhode Island, United States

## Abstract

Using the National Institutes of Health (NIH) Stage Model framework, we are conducting a Stage III/IV embedded trial to evaluate the effects of personalized music on agitated behaviors in nursing home (NH) residents with dementia under two research conditions--less pragmatic, more researcher involvement (Stage III) and more pragmatic, less researcher involvement (Stage IV). We are conducting a three-year trial in 81 NHs, with 27 NHs receiving the intervention per year. Behavior frequency is assessed via resident MDS assessments, staff interviews, and direct observations of residents. During the first year, researchers interview NH staff and observe residents with dementia in 54 randomly selected NHs (27 treatment and 27 control, parallel design). MDS assessments are available for all 81 NHs throughout the three-year study (stepped-wedge design). In the 54 NHs in the parallel design, we compare staff interview and NH-conducted resident assessments and, using multiple imputation methods, we impute staff interview data for eligible residents of all 81 NHs in order to estimate the effect of the intervention under the step-wedge design. There are four key features of this trial: 1) combination of parallel and stepped-wedge designs; 2) equilibrating researcher-collected behavior data to NH-collected behavior data to impute research-collected behavior data for some residents; and 3) simulated resident selection process in control facilities to improve comparisons of effects across treatment groups. This design has the potential to shorten the research timeline by iteratively assessing real-world efficacy and large-scale effectiveness. Our design will inform pragmatic testing of other interventions with limited efficacy evidence.

